# Timing-dependent neuroprotective effects of Virgin coconut oil against chronic restraint stress-induced neurobehavioral and hippocampal dysfunction: GC-MS profiling and *in vivo* evaluation

**DOI:** 10.3389/fnut.2026.1936663

**Published:** 2026-10-02

**Authors:** Rucha Sahebrao Dafale, Gitika Bhasin, Shobha U. Kamath, Ujjal Bose, Ramesh Babu MG, Vasanthalaxmi K, Dhiren Punja, Manjula SD

**Affiliations:** 1 Department of Physiology, Kasturba Medical College, Manipal Academy of Higher Education, Manipal, India; 2 Division of Yoga, Center of Integrative Medicine and Research, Manipal Academy of Higher Education, Manipal, India; 3 Department of Biochemistry, Kasturba Medical College, Manipal Academy of Higher Education, Manipal, India; 4 Department of Pharmacology, College of Medicine, American University of Antigua, Coolidge, Antigua and Barbuda; 5 Department of Basic Medical Sciences, Manipal Academy of Higher Education, Manipal, India

**Keywords:** chronic restraint stress, neurobehavioral, nutraceutical, nutritional neuroprotection, oxidative stress, phytochemical profiling, virgin coconut oil

## Abstract

**Background:**

Chronic stress, often exacerbated by improper lifestyle, long-term physical confinement, and emotional distress, these are the major contributor to neuropsychiatric disorders, mostly through hippocampal dysfunction. Though synthetic drugs offer symptomatic relief, their side effects prompt a growing interest in natural plant-derived nutraceuticals. Virgin coconut oil (VCO), rich in medium-chain fatty acids, polyphenols, and antioxidants, has demonstrated preliminary neuroprotective activity; however, its pharmacological basis in hippocampal dysfunction induced by chronic restraint stress (CRS) remains inadequately characterized. Herein, we investigated the neuroprotective activity of VCO by administering it in a time-staggered manner (Pre-Treatment, Co-treatment, and Post-Treatment) and assessed its effects through physiological, behavioral, antioxidant, and histopathological analyses. Our secondary objective was to profile the phytoconstituents of VCO using Gas Chromatography-Mass Spectrometry (GC-MS).

**Methods:**

Adult male Wistar rats were randomized into six groups (*n* = 6): Control, CRS, VCO-alone, Pre-VCO, Co-VCO, and Post-VCO. VCO was administered in a time-staggered manner to delineate its prophylactic, adjuvant, and therapeutic potential. Animals underwent physiological, behavioral (Sucrose Preference Test, Elevated Plus Maze, radial arm maze), biochemical (corticosterone, lipid peroxidation, glutathione, superoxide dismutase, catalase), and histopathological assessments (Nissl staining). Lastly, we performed phytochemical characterization using Gas Chromatography-Mass Spectrometry (GC-MS).

**Results:**

VCO significantly enhanced spatial learning and memory while attenuating anhedonia and anxiety-like behavior. Biochemically, VCO reduced corticosterone and lipid peroxidation levels, restored antioxidant markers, and reinstated hippocampal neuronal morphology, collectively indicating modulation of neurobehavioral activity and restoration of redox homeostasis. Pre-VCO demonstrated superior neuroprotection, suggesting stronger prophylactic and adjunctive pharmacological activity. GC-MS identified caprylic acid possessing the most abundant chromatographic peak alongside polyphenolic compounds, supporting a plausible mechanistic basis for the neuroprotective effects of VCO.

**Conclusion:**

VCO demonstrates significantly higher neuroprotective efficacy when administered as Pre-treatment in CRS rats. Overall, these findings establish VCO as a dietary functional food with measurable prophylactic and adjunctive neuroprotective efficacy. These may plausibly be due to the major content of the MCFA-like caprylic acid in VCO mitigating oxidative stress and hippocampal neuronal dysfunction. This study provides GC-MS-based compositional evidence and *in vivo* functional validation supporting VCO's translational potential as a dietary strategy to mitigate the chronic stress-associated neuropsychiatric burden in human populations.

## Graphical Abstract

**Figure:**
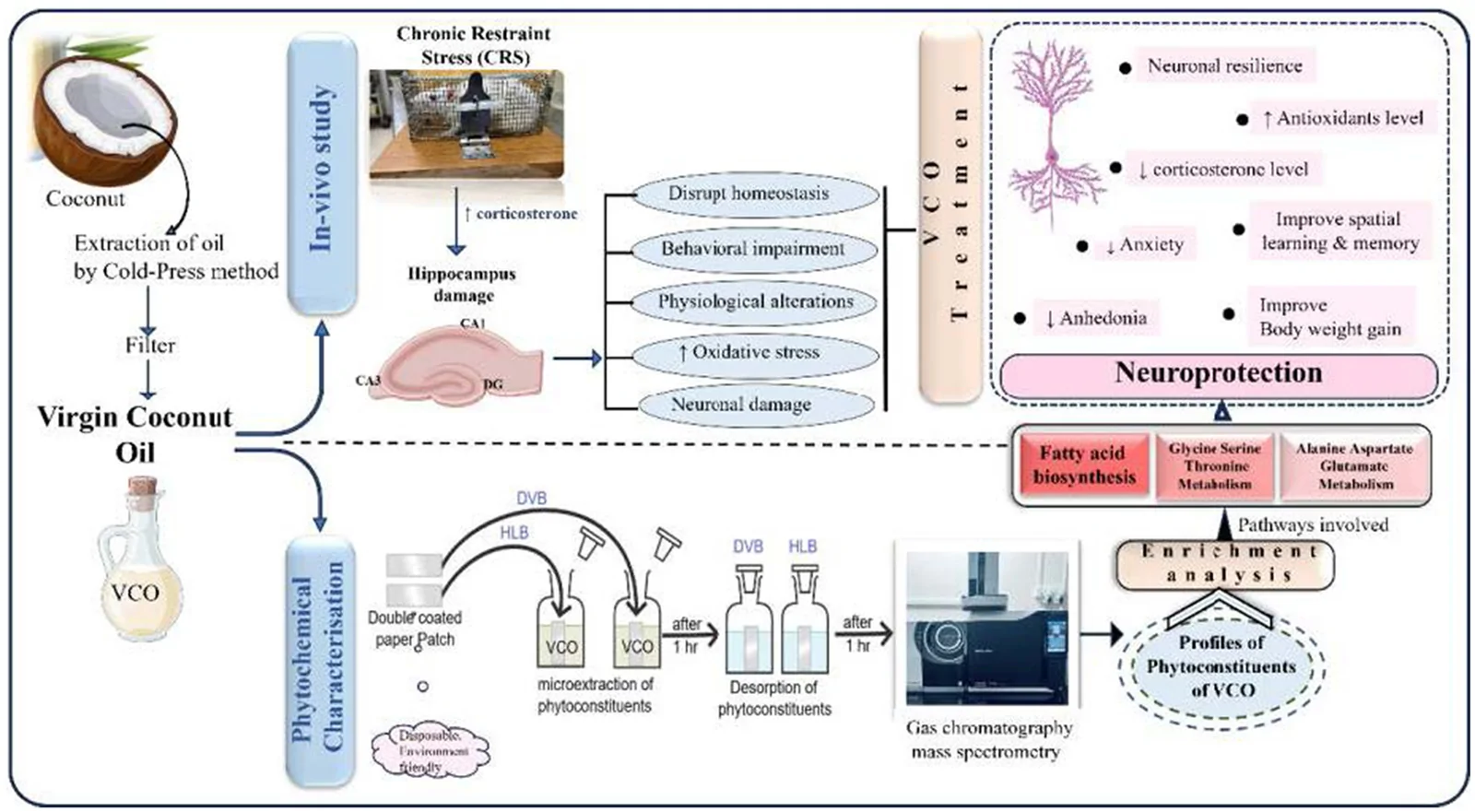

## 1 Introduction

Several studies theorized that stress is one of the significant environmental factors that impact individuals' physical and psychological health and bring about mental illness (1). Today's world is facing a major challenge of prolonged physical confinement and emotional distress. It is stated that prolonged immobilization for more than 30 years is equal to multiple restraint stress sessions, which can reportedly cause stress and hinder the functioning of the brain, which may give rise to multiple neuropsychiatric disorders (2, 3). Prolonged immobilization with stress is associated with morphological and functional variations of synapses, neural circuits, and neurotransmitters in several compartments of the brain (4). It has been well accepted that acute or chronic stress is the key pathogenesis for the onset of psychiatric disorders (5). However, depending on the severity and the duration of exposure, the progression of psychiatric disorders takes place. Among the brain regions most vulnerable to chronic stress is the hippocampus (6, 7). This causes impairment of emotional response, cognition, and memory (8).

For the past two decades, oral monoamine oxidase inhibitors, serotonin reuptake inhibitors (SSRIs), and tricyclic antidepressants (TCAs) have been among potential treatment for attenuating chronic stress-associated neuropsychiatric disorders (9). It is worth noting that only one-third of antidepressant recipients respond to the first antidepressant medicine. Unfortunately, more than one-third of the patients may initially respond to treatment due to drug-placebo differences and later develop treatment-resistant depression (10, 11). The current synthetic drug for chronic stress management displays an unmet therapeutic need in terms of drug safety and efficacy. For example, fluoxetine is known for its quick action, but research has disclosed that long-term usage could adversely affect reproductive health and can lead to gut issues (12). Despite this, well-characterized conventional pharmacological management remains limited by adverse effect profiles and tolerability concerns. Collectively encouraging a rising scientific and consumer interest in natural, safe, effective neuroprotective strategies. In that context, functional foods or food-derived moieties are widely adopted by an extensive population owing to their accessibility and biocompatibility.

Nutraceuticals refer to nutrition-rich food-based medicines obtained from derivatives or from whole foods. It has multifunctional activity plays a nutritional and therapeutic role, making them more advantageous than synthetic drugs. We found a nutraceutical, Virgin Coconut Oil (VCO), which is one of the potent edible oils abundantly available and used widely in major parts of Asia. It is a functional food traditionally used for cooking in various cuisines (13). It is one of the pharmacologically active nutraceuticals used for treating skin, hair and brain nourishment (14). Pharmacologically, VCO possesses antioxidant, anti-diabetic, anti-inflammatory, anti-oxidative, anti-cancer, and neuroprotective properties (15). The promising properties of VCO draw our attention to expand its neurotherapeutic functionality in stress-related abnormalities and disorders.

To provide value addition to VCO and expand its translational outcomes, it is of paramount importance to evaluate VCO's potency. In the process of drug development, preclinical research (animal models) can be a good choice for targeting the clinical translation of research (16). Rodent models are highly acceptable models in preclinical research. Considering the global burden of young adults facing, a prolong physical confinement, and emotional distress causing psychiatric disorders can be reproduced at its best in the chronic restraint stress (CRS) model of rats. Chronic restraint stress is a well-established preclinical paradigm in which involuntary physical confinement induces acute emotional distress through loss of behavioral control and fear, triggering immediate sympathoadrenal activation (17). This is characterized by surges in plasma catecholamines, tachycardia, hypertension and stress-induced hyperthermia (18), together with robust activation of the hypothalamic–pituitary–adrenal (HPA) axis and sustained corticosterone release (19). Repeated exposure to this paradigm over weeks produces measurable neurobehavioral and structural changes, particularly within stress-sensitive limbic regions such as the hippocampus (20). CRS is a stress model that includes a predictable stressor (i.e., immobilization); therefore, it affects the brain differently from other stress models such as chronic unpredictable mild stress (CUMS), learned helplessness, and social defeat, which comprise unpredictable stressors (21). To date, VCO has demonstrated a positive outcome against CUMS, post-traumatic stress disorder, and other neuronal disorders, indicating its neuroprotective efficiency with negligible toxicity (22, 23). So far, VCO's activity is rarely known in the chronic restraint stress (CRS) model of rats. This makes it crucial to investigate how VCO behaves in different stress models. Thus, examining VCO's potency in CRS rats might be intriguing, as it directly addresses the underexplored gap in validating VCO's neuroprotective effects against CRS-induced hippocampal dysfunction- a prevalent yet understudied hallmark of chronic stress-related psychiatric disorders.

Given these facts, we hypothesize that VCO may mitigate the deleterious effects of CRS in adult male rats. The current study primarily aims to ascertain a systematic understanding of the neuroprotective potency of VCO. Here, we administered VCO in a time-staggered manner (Pre-Treatment, Co-treatment, and Post-Treatment of VCO) in CRS rats. The neurotherapeutic potential was evaluated through physiological, behavioral, biochemical, and histopathological analyses. Our secondary objective was to profile the phytoconstituents of VCO using Gas Chromatography-Mass Spectrometry (GC-MS). This strategy will also assist in understanding time-dependent variation and elucidating the possible mechanistic approach of VCO in mitigating the CRS-induced neurodisturbance. Also, this approach will provide a robust foundation for navigating future clinical translation.

From a nutritional perspective, this study has direct relevance to the growing scientific discourse on nutraceuticals and dietary bioactive compounds as modulators of neuroendocrine stress responses. This supports the positioning of VCO within a nutraceutical framework for stress-resilient dietary strategies.

## 2 Materials and methods

### 2.1 Cold-pressed Virgin coconut oil (VCO)

VCO was obtained from coconuts, sourced from the coastal region of Udupi, Karnataka, India and authenticated by a botanist. The oil was extracted from the white part of the coconut fruit via a cold-pressed technique. The coconuts were finely grated, and the milk was extracted, as fine grating yields more oil. The milk was collected and allowed to sit undisturbed for 24–48 h. This allows the oil to separate in a density gradient manner. The solids and milk sank to the bottom, and the oil started floating on top. The oil was carefully collected from the top and filtered using a sieve (24–26). This cold-pressed technique relies on a natural fermentation process to break the oil-water bond. This allows the milk to demulsify into three layers: oil (top), cream (middle-protein), and water (bottom). This technique does not involve chemicals or heat, hence known as the cold-pressed technique and relies on natural gravitational separation. It is a time-consuming and low-yield process; nevertheless, more advantageous for retaining the phytoconstituents of VCO, like polyphenols and antioxidants (25, 26).

The prescribed condition limits oxidative degradation and keeps the content of VCO stable. In the current study, we administered VCO at a dose of 5 ml/kg body weight of rats daily for 21 days, which was verified in our previous pilot study (27). The same VCO was used for GC-MS analysis.

### 2.2 Gas-chromatography-mass-spectrometry

The Phytochemical profiling of VCO was carried out using gas chromatography-mass spectrometry (GC-MS) (GC/MS-QP2020 NX SHIMADZU, Shimadzu Corp., Tokyo, Japan) according to the previously published protocol of Poojary et al. (detailed procedure attached in Supplementary File). First, we performed thin-film solid-phase microextraction (TF-SPE) using two different disposable paper-based patches: one double-coated with Divinylbenzene (DVB) and another double-coated with a hydrophilic-lipophilic balanced (HLB) polymer (28–30). Next, the DVB and HLB patches were allowed to absorb the VCO. The patch was later transferred to acetonitrile for the desorption process. This process allows VCO metabolites to be desorbed in acetonitrile carried by the patch. Lastly, 1 mL of acetonitrile was sampled on a GC-MS instrument equipped with a wax column. For the detection of spectra, an ionization energy method was utilized in which a high ionization energy was applied. The obtained metabolomic data were compared with the National Institute of Standards and Technology (NIST) library based on Component area, retention time, and mass spectral pattern for the profiling of phytoconstituents (31, 32). Further, the metabolomic data were used to explore the probable mechanistic pathway by performing enrichment analysis using MetaboAnalyst 6.0 software.

### 2.3 *In-vivo* study design

The *in-vivo* study begins with grouping and labeling by randomization method for the allocation and, cage composition (3 animals/cage, with cages rotated across shelf positions to offset location effects). Animals were assigned into 6 different groups (*n* = 6): Control, CRS, only VCO, Pre-VCO, Co-VCO, Post-VCO, and kept for 7–10 days for acclimatization. The sample size of *n* = 6 animals per group was selected based on established convention in preclinical animal nutrition/toxicology research and our pilot study, where *n* = 6 is widely regarded as the minimum group size required to achieve adequate statistical power for parametric analyses while adhering to the 3Rs principle (Replacement, Reduction, Refinement) of minimizing animal use, as endorsed by NC3Rs and required by our institutional Animal Ethics Committee. Animals were dosed as shown in Figure 1, and from the next day of dosing, animals were exposed to behavioral evaluation. Lastly, Animals were deeply anesthetized, blood was withdrawn by terminal cardiac puncture under anesthesia, and decapitation followed to allow rapid dissection of brain and adrenal tissue. Next, the whole brain was removed, and one hemisphere was excised and transferred to formalin solution for histopathological evaluation. From the other hemisphere, the hippocampus was excised and snap-frozen in liquid nitrogen, and further used for biochemical evaluation.

**Figure 1:**
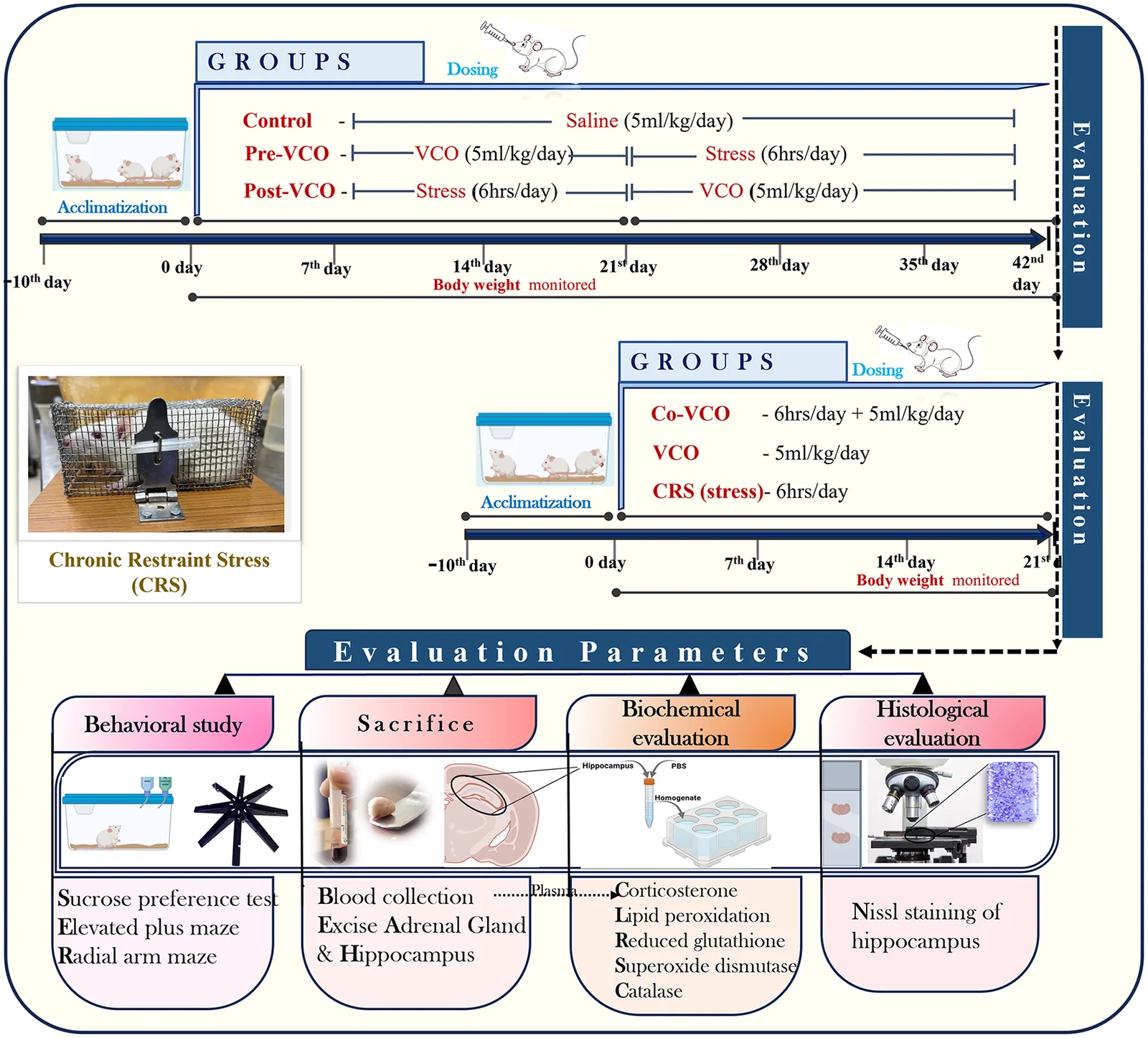
Methodology implemented for investigating the neuroprotective effect of VCO in an *in-vivo* study.

### 2.4 Animals and ethical statement

In the current study, 3-month-old age 36 adult male albino Wistar rats, weighing between 200 and 250 g, were issued from “Central Animal Research Facility, Manipal.” Animals were housed in controlled conditions at a temperature of 22 ± 1 °C, humidity (60% ± 5%, and a 12 h-light/12 h-dark cycle. The animals were housed in a husk-bedded polypropylene cage. All animals had free access to a standard diet and water *ad libitum*. All experimental procedures were carried out under the regulation of the “Committee for Control and Supervision of Experiments on Animals” (CPCSEA). For this research, animals were sanctioned by the “Institutional Animal Ethics Committee (IAEC) (registration no. 94/PO/ReBi/99/CPCSEA) of Kasturba Medical College, MAHE, Manipal. Ethical clearance no. (IAEC/KMC/61/2022).

### 2.5 Stress induction: chronic restraint stress (CRS) model

The restrainer was constructed according to Aalling et al. (33) with a few modifications. The base of the restrainer was made of wood, and the upper part was covered with iron wire mesh, with dimensions of 13 cm × 3 cm × 3 cm (L × W × H) in a rectangular shape. The restrainer was designed in such a way that animals were immobilized with a provision of ventilation. In this study, we immobilized the animals in a restrainer daily for 21 days (6 h/day) to induce CRS, without food and water (33).

### 2.6 Physiological evaluation

#### 2.6.1 Body weight

One of the important physiological parameters is a change in body weight. In the current study, the body weight of the animal was monitored twice a week throughout the study. To determine the effect of VCO treatment on body weight gain, we considered the effective period of the last 21 days for every group to calculate the weight gain using the following formula.


Weight gain (%) =Weight gain (Final body weight−initial body weight at day 0) (g) / weight at Day 0 (g)×100

#### 2.6.2 Adrenal gland weight

After completion of the last behavioral session, animals were sacrificed, their adrenal glands were excised, and weight was measured. To preclude variation in left and right adrenal glands, a relative weight was calculated.


$$\begin{array}{l} Mean\;adrenal\;gland\;weight\;\left(g\right)=Left\;adrenal\;+Right\;adrenal\;weight/\;2 \end{array}$$

%Relative Adrenal Weight =mean adrenal gland weight (g)/Body Weight (g)× 100

### 2.7 Behavioral study

#### 2.7.1 Sucrose preference test

In this study, SPT was performed to ascertain the effect of VCO on the anhedonia of rats according to Sun et al. (34). In this study, SPT was executed on all six groups to ascertain the effect of VCO on the anhedonia of rats. These animals were offered 2 bottles of 1% sucrose water for 24 h in an individual cage. Next, animals were food- and water-deprived for 23 h. Then one of the two bottles was replaced with water for 1 h (Supplementary Figure 1). The difference in sucrose intake between the groups was noted and computed using the formula (34).


Sucrose preference (%)=consumption of sucrose (ml)/Total sucrose consumed (ml)× 100

#### 2.7.2 Elevated plus maze

EPM is an apparatus usually used to evaluate rodents' behavioral activities, such as the anxiety index. In the current study, we performed the EPM test according to Sharma A.C. The behavioral videos were recorded and scored by an observer blind to group allocation. The EPM is completely black (inside and outside) and consists of four arms in a plus shape at a height of 40 cm from the ground, as shown in Supplementary Figure 2. Two closed arms covered with walls are oppositely placed, and the other two are open without walls. The four arms are connected at a central square of 10 cm^2^. At the beginning, animals were placed at the end of the arm, facing toward the central square on EPM. The rats were allowed to discover the EPM for 5 min. The anxiety-related behavior was assessed by counting (i) the number of open arm (OA) entries, (ii) No. of closed arm (CA) entries, (iii) time spent in OA, (iv) time spent in CA, and (v) anxiety index (35).


$$\begin{array}{l} \text{Percent time spent in OAs }\left(\text{\%}\right)=\text{time spent in OAs }\left(\text{s}\right)/300\left(\text{s}\right)\times \;100 \end{array}$$

Anxiety Index =1−[(time spent in OAs/total test duration)+(OA entries/(total OA entries+total CA entries))/ 2]

#### 2.7.3 Radial arm maze

To evaluate the spatial learning, memory, and cognition of the animal, we performed the radial arm maze test (RAM). To track the spatial learning and memory of animals, a video camera was placed above the apparatus and behavioral videos were scored by an observer blind to group allocation. The RAM test was performed according to Togo et al., after CRS and treatment protocol, with some modifications. Before the behavioral study began, all animals were housed individually and kept on food and water deprivation until they reached 85% of their initial body weight. The radial arm maze setup consisted of eight channels of 25 cm diameter around the center, and the wall space was 65 cm (Supplementary Figure 3). The behavioral analysis of RAM includes 3 phases: Habituation, Acquisition, and Retention. Initially, all three groups were subjected to a radial arm maze for Habituation (one session each day for 3 days) and continued the same for seven consecutive days as the acquisition phase. To ensure that the rat had acquired the skill to search for the entire length of each arm, a food reward was shifted toward the end of each session. Later, each rat was permitted 10 min to consume the rewards from all eight arms.

Radial arm maze testing was conducted for seven consecutive days; each day one trial was carried out. Later, each rat was allowed 10 min to catch the reward from all arms or until the animals reached 85% of correct choice entries. During each trial, the food reward was kept toward the end of each arm to confirm that the rat had no vicinity to see the food reward from the center of the platform. Food was baited in four arms each time out of eight, following the arm sequence of 1, 4, 5, and 7 and maintaining the same order each time. At the beginning of each trial, the animal was placed on the center platform and was allowed to collect the food reward for 10 min. After consuming all four rewards, the animals were returned to their home cage. After completing each behavioral session, animals were provided with standard food pellets at a rate of 12% to 16% of their body weight. Upon completion of each trial, the radial arm maze chambers were sterilized with ethanol. Retention memory was studied 7 days after the last day of the acquisition phase; in the period in between, animals were housed back in a cage.

The study was scored using various parameters: (i) Time to complete the task (TCT) (retrieval latency), (ii) % Correct choice entries (%CCE) - first entry into a previously unvisited baited arm within a trial, %CCE = (number of correct first entries into baited arms / total baited arms) × 100, (iii) No. of error entries in the acquisition phase and retention phase [reference memory error (RME) and working memory error (WME)] (27, 36). “Reference memory errors” (RME) were defined as entry into an unbaited arm, requiring animals to remember from earlier days which arms were unbaited to avoid error. While the “Working memory errors” (WME) were scored as re-entries into an arm from which food had already been consumed. Every parameter was evaluated in the acquisition and retention phase.

### 2.8 Biochemical evaluation

#### 2.8.1 Specimen preparation

The biochemical analysis was performed using tissue homogenate. The snap-frozen hippocampal tissue was used to prepare a tissue homogenate. The tissue was placed in ice-cold isotonic saline after being removed from a −80 °C refrigerator. The tissue was homogenized in 10% (w/v) Sodium Phosphate buffer solution (0.1 M, pH 7.4) in an ice bath. Next, the prepared mixture was centrifuged at 4 °C, 10,000 rpm for 15 mins. The supernatant was separated and stored at −20 °C and was utilized for all biochemical analysis except corticosterone analysis, for corticosterone analysis plasma obtained from animal blood was used. All the samples were tested in duplicates for every biochemical parameter (37).

#### 2.8.2 Corticosterone (CORT)

The plasma obtained from the rat blood was used to estimate CORT levels via the competitive ELISA method using a Kit (E-EL-0160; Elabscience Biotechnology Inc., Houston, TX, USA) according to the manufacturer's protocol. The color change was recorded at 430 nm using a spectrophotometer. The absorbance values were expressed in μg/dl of plasma (38).

### 2.9 Oxidative stress biomarkers

#### 2.9.1 Reduced glutathione (GSH)

GSH was measured using Elman's method. The tissue homogenate was mixed with trichloroacetic acid and DTNB, and absorbance was taken at 412 nm using a UV spectrophotometer. The GSH concentrations were calculated as μmol/ mg of tissue (39).

#### 2.9.2 Lipid peroxidation

We measured MDA using the thiobarbituric acid method according to Wills et al. (1996). The final absorbance was recorded at 532 nm using a UV spectrophotometer. The concentrations of MDA were expressed as nmol/mg of tissue (38).

#### 2.9.3 Superoxide dismutase (SOD)

The SOD level of hippocampal tissue was estimated according to the Misra and Fridovich protocol. At the end, the solution's absorbance was taken at 480 nm using a UV spectrophotometer at 15-s intervals for 2 min. Each unit of SOD enzyme was required for 50% inhibition of oxidation. The values were expressed in U/mg of tissue (38).

#### 2.9.4 Catalase (CAT)

CAT level was estimated using the previous protocol of Sinha (1972). The tissue homogenate was mixed with phosphate buffer treated with hydrogen peroxide and dichromate solution. The absorbance was taken at 570 nm. CAT activity was expressed as micromoles of H_2_O_2_ consumed/min/mg of tissue (38).

### 2.10 Histopathological study

#### 2.10.1 Nissl staining

The excised half a hemisphere of the brain of the animal was post-fixed in 10% formalin. The tissues were embedded in paraffin, and solidified blocks were made. Next, the coronal sections were acquired using a Leica semiautomated microtome at 20 μm thickness. The sections were mounted on albumin-coated slides and dried on a slide warmer. Later, the sections of the hippocampus of the brain were stained using the Nissl staining technique. The procedure was standardized and performed according to Atiba et al. (40). Finally, the slides were mounted using DPX (Dibutylphthalate Polystyrene Xylene) mountant and secured with a coverslip. For histological evaluation were coded before imaging and captured by the observer blind to group allocation. Brain slides were examined under an Olympus microscope camera, and images of the hippocampus were captured. We focused on different regions of the hippocampus: dentate gyrus (DG), cornu ammonis 3 (CA3) and cornu ammonis 1 (CA1) within the field of view (40).

#### 2.10.2 H & E staining

The H & E staining was performed to evaluate the morphometric changes in the adrenal gland. The excised tissue of the adrenal gland was post-fixed in 10% formalin and processed for paraffinization to form a solid block. Further, sections of 20 μm thickness were acquired and stained with H & E according to the procedure depicted by Li et al. (41). Finally, the section was dried and sealed with DPX and a cover slip. The histological sections were coded before imaging and captured by the observer blind to group allocation. The images were captured from the slide within the field of view of the microscope at 10 × magnification.

### 2.11 Statistical analysis

To analyze the *in-vivo* data, GraphPad Prism Version 10 was used. For physiological, behavioral, and biochemical data, For Body weight gain, % correct choice entries, working memory error, and reference memory error, we performed Two-way ANOVA with a Šídák *post-hoc* test. For the remaining parameters, One-way analysis of variance (ANOVA) with Šídák *post-hoc* was applied. This unified model simultaneously partitions total variance and effectively controls the family-wise Type I error rate. Because the specialized temporal relationships of the treatment arms (pre-, co-, and post-treatment) created a structural design with unobserved factor combinations, groups were analyzed using a unified One-Way Analysis of Variance (ANOVA) framework across all six experimental conditions. To perform targeted, statistically rigorous inter-group comparisons without over-inflating the false-positive rate, a Šídák's multiple comparisons test was applied.

Values are expressed in mean ± standard error and considered statistically significant at 0.05 (*p*-*value* of less than 0.05 (*p* < 0.05) for each parameter.

## 3 Results

### 3.1 Phytochemical profiling of VCO using GC-MS

To elucidate the phytoconstituents of VCO, we performed untargeted GC-MS analysis in triplicate, and the stable pattern of fatty acids was checked for the quality of the experimental batch (42, 43). The chromatogram of VCO extracted from the DVB (affinity toward moderately polar metabolites) and hydrophilic-lipophilic balance (HLB) (affinity toward polar and non-polar metabolites) patch was recorded. The chromatogram obtained was compared with the standard NIST library, using the retention time of each metabolite. From the pool of metabolites (attached in the Supplementary Tables 1, 2), we selected the top bioactive metabolites that may contribute to exerting pharmacological activity. As shown in Figure 2C, DVB and HLB share 32.5% common phytoconstituents, while DVB and HLB exhibit 37.2% and 30.2% variance in phytoconstituents. The Variance in phytoconstituents exhibits differential affinity of the metabolite, thus assisting in the complete profiling of VCO constituents. This differential approach reveals the polar and non-polar affinities of the phytoconstituents and aids in selecting the appropriate method for targeted phytoconstituent analysis. Our data indicate the presence of medium-chain fatty acids in both DVB and HLB. The highest peak area detected in HLB was of caprylic acid (RT-21.66). In DVB, the first highest peak noted was of glycerol tricaprylate (RT- 19.44), a derivative of caprylic acid, and the second highest was caprylic acid (Figure 3). In a nutshell, the GC-MS data indicate the abundance of caprylic acid.

**Figure 2:**
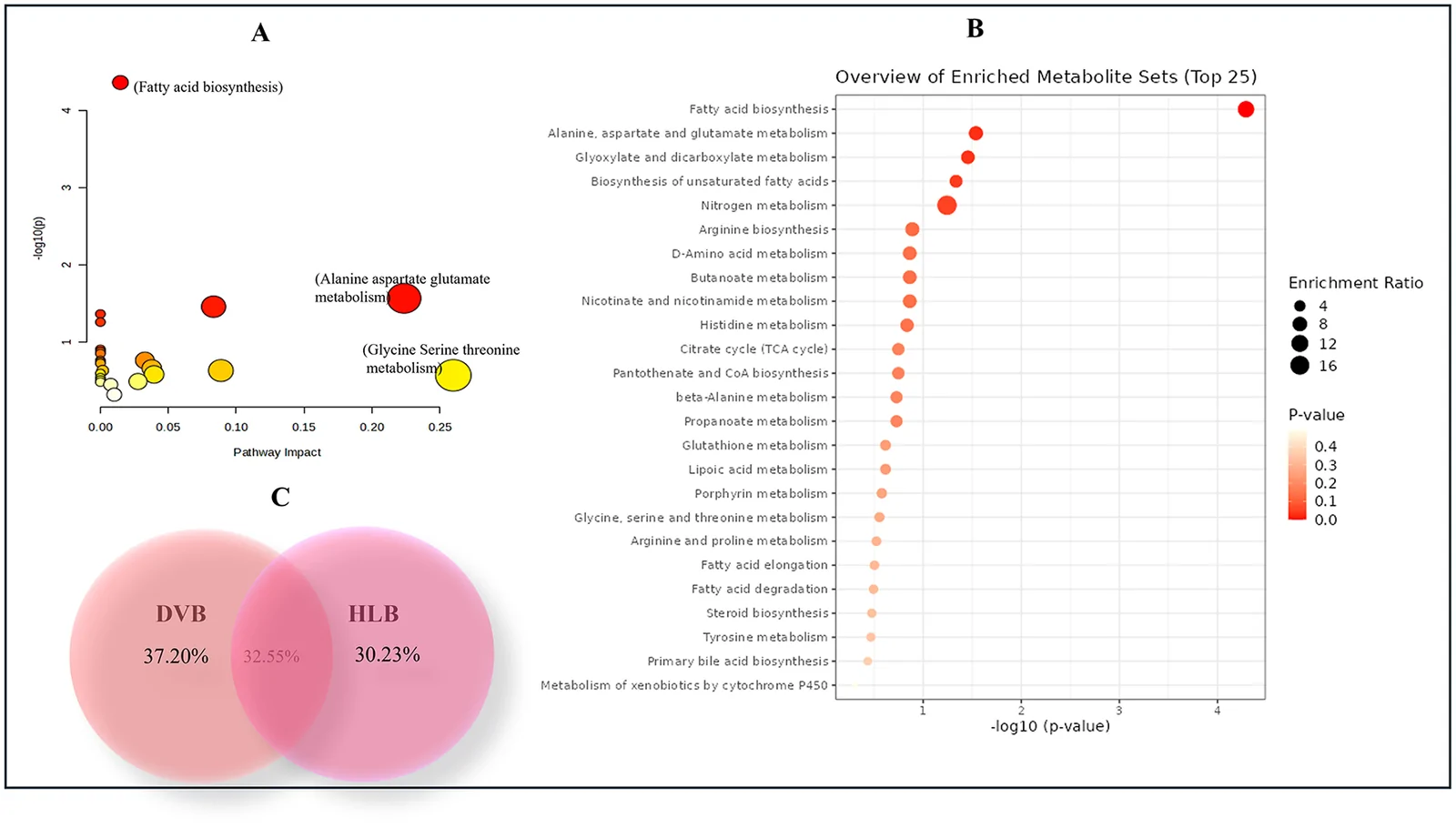
**(A)** GC-MS profiling of VCO showing the impact and significance of metabolites on the pathway. **(B)** Fatty acid biosynthesis shows the highest significance. The metabolites have the highest impact on Glycine serine threonine metabolism, alanine, aspartate, glutamate metabolism pathways. **(C)** Venn diagram of phytocompounds of VCO representing the percentage of common and different metabolites extracted using HLB and DVB patch. (as shown in Supplementary Tables 1, 2).

**Figure 3:**
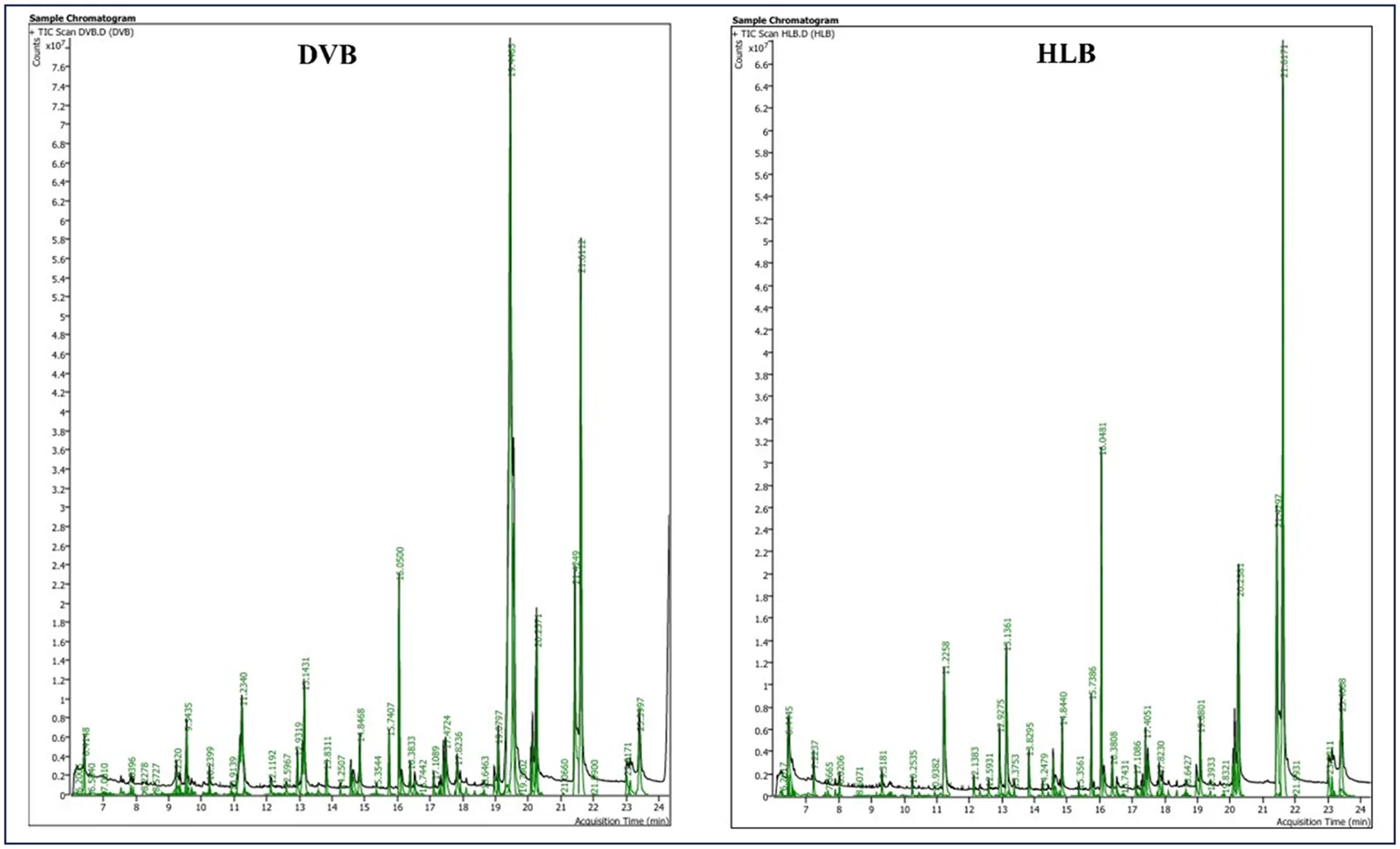
GC-MS chromatogram of phytoconstituents of VCO obtained from HLB and DVB.

The bioactive profiling of the plant extract also helps in elucidating the possible mechanism of action of the drug. Therefore, we used the obtained metabolomic data for screening the pathways. For screening the pathways, we compiled the phytocompound data obtained from the DVB and HLB patches and performed enrichment analysis using MetaboAnalyst 6.0 software. From the results, we found that the phytoconstituents of VCO take part in 25 different pathways. The phytoconstituents showed the highest impact on serine-threonine pathways and the second-highest impact on alanine, aspartate, and glutamate pathways. Phytoconstituents have the highest significance in the fatty acid metabolism pathway, and the maximum contribution was from caprylic acid, capric acid, lauric acid, and palmitic acid (Figures 2A, B).

### 3.2 *In vivo*- pharmacological assessment

#### 3.2.1 Physiological evaluation

##### 3.2.1.1 Body weight change

The effect of VCO on body weight change is depicted in Figure 4a. The body weight gain in CRS animals was decreased compared to control (*p* = 0.004) and only in VCO-treated rats (*p* = 0.009), indicating successful induction of CRS. Animals exposed to CRS and treated with VCO showed improvement in body weight gain. Among VCO-treated groups, the Pre-treatment (*p* < 0.001) group showed the highest body weight gain compared to the Co-treatment (*p* = 0.005) and Post-treatment (*p* = 0.010) groups when compared to CRS. Furthermore, only VCO-treated rats showed no significant effect compared to control rats.

**Figure 4:**
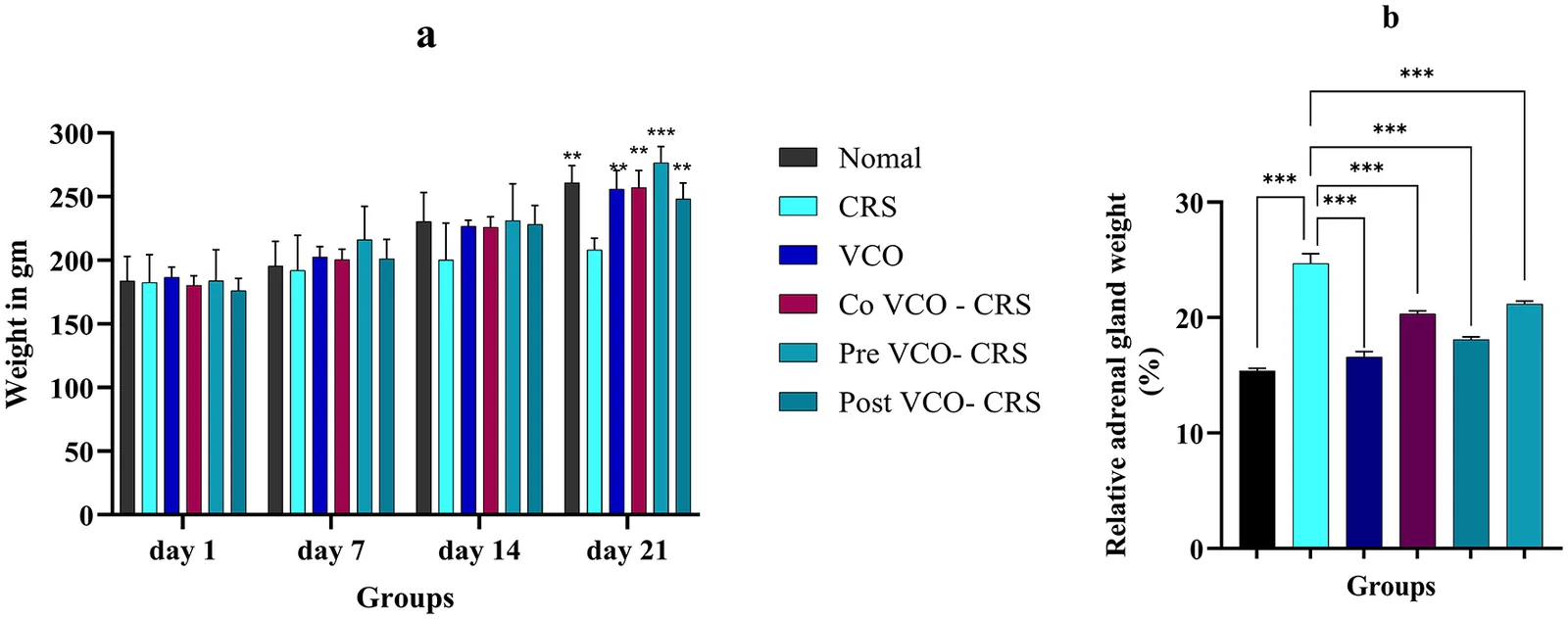
VCO attenuates CRS-induced physiological changes. Body weight gain **(a)** Two-way ANOVA, Relative adrenal gland weight, **(b)** One-way ANOVA with Šídák's multiple comparisons test. All data are represented as mean ± S.E.M. ns (non-significant) *p* > 0.05, *p < 0.05, **p < 0.01, ***p < 0.001.

##### 3.2.1.2 Adrenal gland weight

The change in adrenal gland weight with respect to VCO treatment is presented in Figure 4b. From the histogram, it is evident that adrenal gland weight was significantly elevated in rats exposed to CRS compared to control group rats (*p* < 0.001). Pre-treatment, Co-treatment, and Post-treatment with VCO in animals exposed to CRS showed a significant diminution in adrenal gland weight compared to CRS group (*p* < 0.001), indicating the ameliorative effect against CRS. Thus, animals treated only with VCO showed no significant difference when compared to the control group rats (*p* = 0.071).

#### 3.2.2 Behavioral evaluation

##### 3.2.2.1 Sucrose preference test

In this study, a sucrose preference test was performed to ascertain the anhedonia effect. The results shown in Figure 5a revealed that animals exposed to CRS had higher anhedonia than the VCO-treated and Control groups. Statistically, the CRS group showed a significant decrease in the % sucrose preference compared to the Control group (*p* < 0.001), indicating an anhedonia effect. On the contrary, treatment of VCO showed enhanced % sucrose preference, indicating substantial improvement in anhedonia in all three groups (Pre VCO-CRS (*p* < 0.001), Co VCO-CRS (*p* < 0.001), Post VCO-CRS (*p* < 0.001) compared to the CRS group.

**Figure 5:**
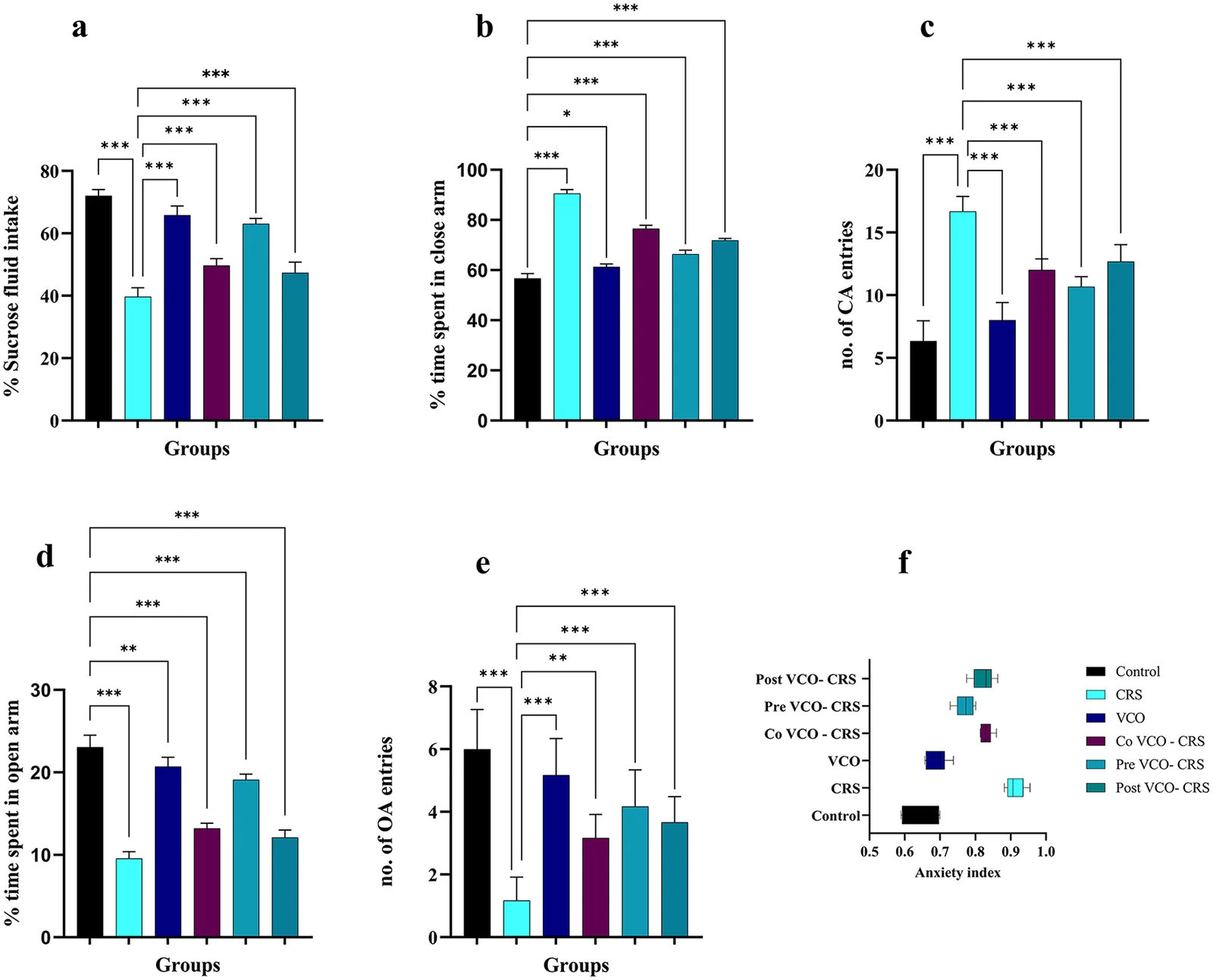
VCO attenuates CRS–induced behavioral changes evaluated using the SPT and EPM—Sucrose preference test **(a)**, % time spent in closed arm **(b)**, No. of closed arm entries **(c)**, % time spent in open arm **(d)**, No. of open arm entries **(e)**, Anxiety index **(f)**. One-way ANOVA with Šídák's multiple comparisons test. All data are represented as mean ± S.E.M. ns (non-significant) *p* > 0.05, ^*^p >0.05, ^**^p > 0.01, ^***^p > 0.001.

##### 3.2.2.2 Elevated plus maze

Chronic stress-induced anxiety-like behavior can be best assessed in the EPM. Herein, the results of VCO treatment on CRS animals are presented in Figure 5. Animals exposed to CRS showed less affinity toward the OA; hence, OA entries and % time spent in the OA were significantly less, while animals preferred to spend more time and enter the closed arm than the control and vice versa in CA. Although animals exposed to CRS and received VCO treatment in a time-staggered manner, Pre-treatment (*p* < 0.001), Co-treatment (*p* < 0.001), and Post-treatment (*p* < 0.001) showed improved preference and affinity to spend more time in OA compared to CRS (Figures 5d, e). More specifically, among VCO-treated animals, Pre-treatment (*p* < 0.001) and Post-treatment (*p* < 0.001) with VCO showed significantly more entries in OA than Co-treatment (*p* < 0.01) when compared to the CRS group animals. On the contrary, it showed a significant reduction in CA entries and % time spent in the closed arm compared to CRS group (*p* < 0.001; Figures 5b, c).

CRS rats exhibited a higher anxiety index than control (*p* < 0.001) and the VCO-treated sham group (*p* < 0.001), while control and only the VCO groups showed no significant change in anxiety index (*p* = 0.06). Overall, the anxiety index was abridged after VCO administration compared to CRS group in Pre-treatment (*p* < 0.001), Co-treatment (*p* < 0.001), and Post-treatment (*p* < 0.001). Comparative changes in time-staggered VCO treatment showed no significant change in Post-treatment compared with Co-treatment and Pre-treatment, while Pre-treatment exhibited a significant difference compared to Co-treatment (*p* = 0.03; Figure 5f).

##### 3.2.2.3 Radial arm maze

The behavioral data point out that, except for CRS animals, all animals passed the minimum criteria of 85% CCE. Control and VCO-only group rats reached the minimum criteria after 4 days in the acquisition phase. The animals received Pre-VCO and Pos-VCO on the 6th day, and Co-treatment was reached by 7th day. However, CRS group animals failed to reach the criteria (Figure 6b).

**Figure 6:**
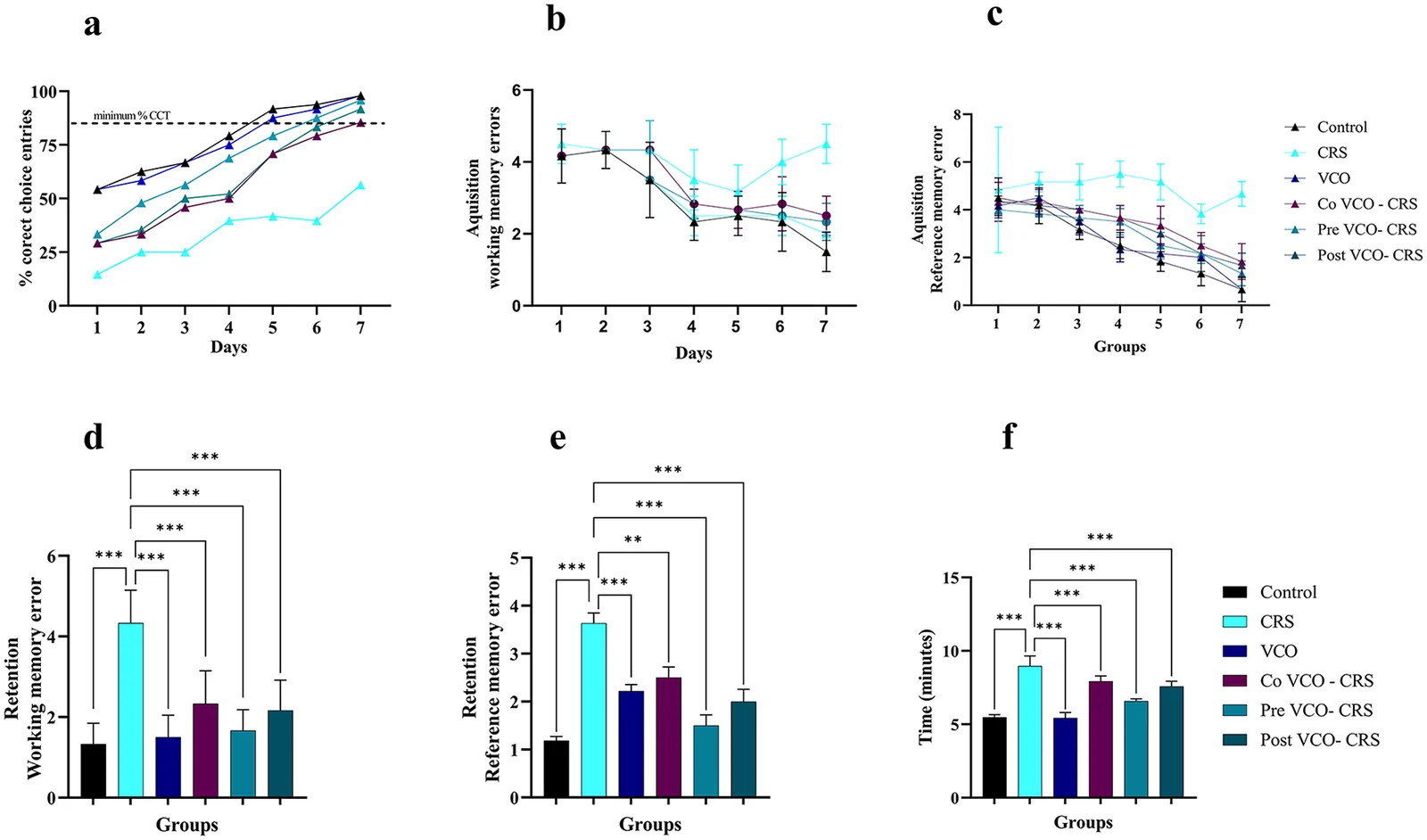
VCO attenuates CRS-induced behavioral changes evaluated using RAM-% correct choice entries **(a)**, acquisition—working memory errors **(b)**, reference memory error **(c)**, retention—working memory error **(d)**, reference memory error **(e)**, time spent to complete the task **(f)**, Two-way ANOVA **(a–c)** and One-way ANOVA **(d–f)** with Šídák's multiple comparisons test. All data are represented as mean ± S.E.M. ns (non-significant) *p* < 0.05, *p < 0.05, **p < 0.01, ***p < 0.001.

Meanwhile, the error entries were increased in CRS animals compared to control animals. The WME and RME were gradually increased in CRS (*p* < 0.001, *p* < 0.001) rats compared to control and only VCO rats. However, VCO treatment reduced the WME and RME in Pre-VCO (*p* < 0.001, *p* < 0.001), Co-VCO (*p* < 0.001, *p* < 0.01), and Post-VCO (*p* < 0.001, *p* < 0.001) groups compared to the CRS group. This signifies that VCO treatment enhanced the learning ability of the animal (Figures 6c, d). Furthermore, to assess the recall ability of the learned task, we performed the task in the retention phase after 7 days. The memory was retained in VCO-treated animals and vice versa in CRS animals. CRS animals attempted more WME and RME than control animals and only VCO animals. The animals treated with VCO to Pre-VCO (*p* < 0.001, *p* < 0.001), Co-VCO (*p* < 0.001, *p* < 0.01), and Post-VCO (*p* < 0.001, *p* < 0.001) groups attempted fewer error entries compared to CRS group (Figures 6e, f).

Hence, CRS animals took more TCT compared to control (*p* < 0.001) animals. However, VCO enhanced the spatial learning and memory ability of animals, hence spent less TCT in Pre-VCO (*p* < 0.001), Co-VCO (*p* < 0.01), and Post-VCO (*p* < 0.001; Figure 6a). This indicates that the CRS rats failed to identify and memorize the clues. The ability of learning and memory was found to be reduced in the RAM task. However, VCO treatment attenuated the impaired spatial learning and memory.

#### 3.2.3 Biochemical evaluation

##### 3.2.3.1 Corticosterone level

Figure 7a represents the level of CORT modified after VCO treatment. In our study, an elevated CORT level was observed in CRS animals' blood plasma (*p* < 0.001). While the treatment of VCO to Pre-VCO (*p* < 0.001), Co-VCO (*p* = 0.002), and Post-VCO (*p* < 0.001) groups abridged the level of CORT by 38.55%, 21.33%, and 25.56%, respectively, compared to the CRS group. No significant change was noted in the VCO group when compared to the control group (*p* = 0.10). The effect of time-staggered administration of VCO in different groups showed that Pre-treatment exhibited (*p* < 0.001) significant reduction in CORT level compared to Co-treatment and Post-treatment, while Co-treatment exhibited *p* = 0.03 significance compared to Post-treatment. These suggest that VCO ameliorates the CRS impact by reducing the CORT level.

**Figure 7:**
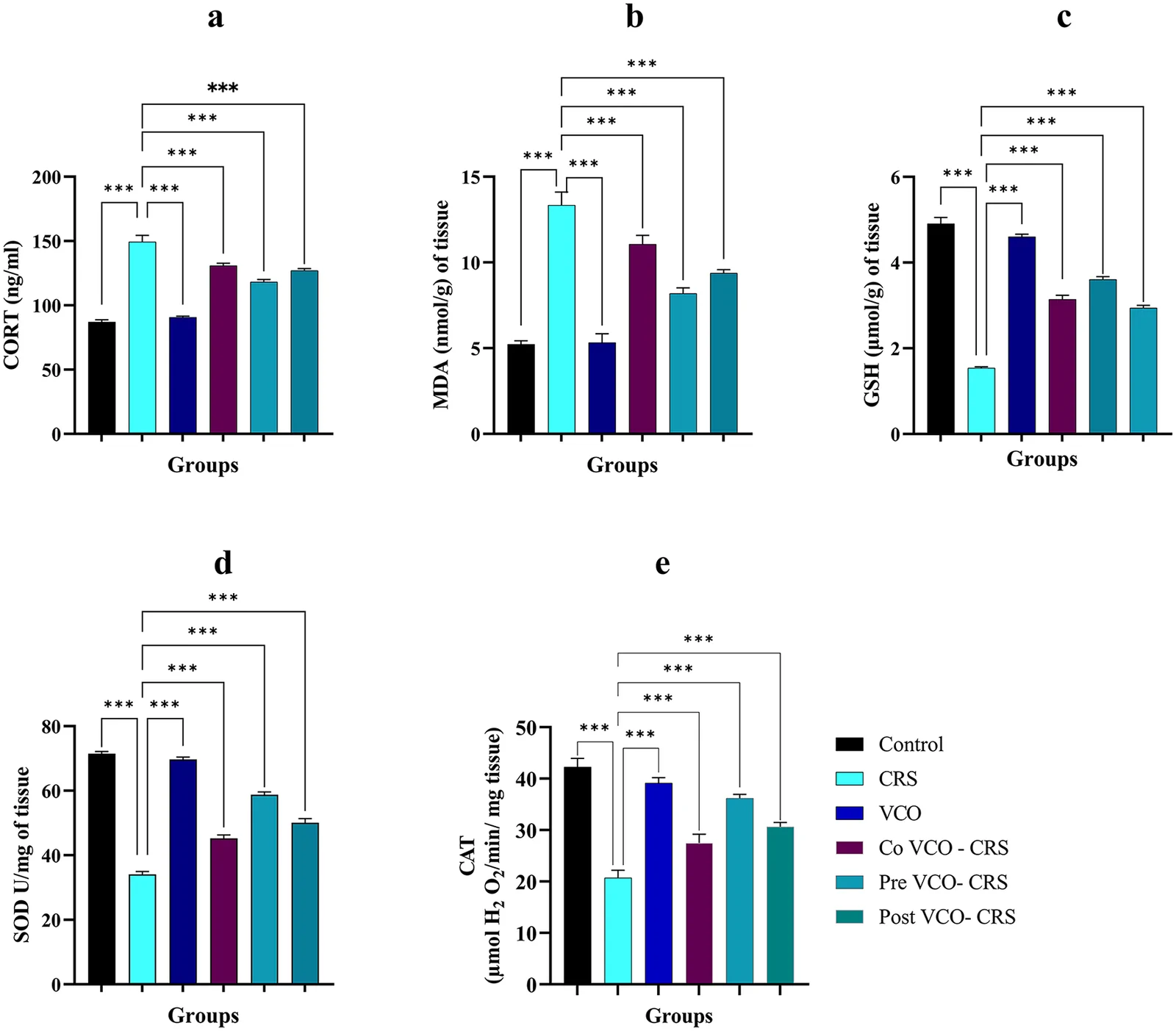
VCO attenuates CRS-induced biochemical alterations: Corticosterone (CORT) **(a)**, Lipid peroxidation (MDA) **(b)**, Reduced Glutathione (GSH) **(c)**, Superoxide Dismutase (SOD) **(d)**, Catalase (CAT) **(e)**. One-way ANOVA with Šídák's multiple comparisons test. All data are represented as mean ± S.E.M. ns (non-significant) *p* < 0.05, *p < 0.05, **p < 0.01, ***p < 0.001.

##### 3.2.3.2 Reduced glutathione level

The results represented in Figure 7c indicate that the level of GSH was depleted in CRS rats compared to control rats (*p* < 0.001). On the other hand, the animals that received only VCO showed no significant change compared to the control. An augmented level of GSH was noted in VCO-treated animals of Pre-VCO, Co-VCO, and Post-VCO groups compared to stress rats, indicating improved antioxidant status in the hippocampus of the brain. Between the treatment groups, Pre-treatment exhibited (*p* < 0.001) a significant elevation of GSH level compared to Co-treatment and Post-treatment, while Co-treatment exhibited *p* < 0.002 significance compared to Post-treatment.

##### 3.2.3.3 Lipid peroxidation level

Increased oxidative stress in CRS condition increases lipid peroxidation, which causes an augmentation of the MDA level. Herein, the histogram presented in Figure 7b delineates an elevated level of MDA in CRS animals compared to the control group (*p* < 0.001). While no significant change was depicted in only VCO group compared to the control group. Although treatment of VCO in Pre-VCO (*p* < 0.001), Co-VCO (*p* < 0.001), and Post-VCO (*p* < 0.001) groups showed a significant change compared to CRS group. In between the treatment groups, Pre-treatment exhibited (*p* < 0.001) a significant reduction of MDA level compared to Co-treatment and Post-treatment, while Pre-treatment exhibited *p* < 0.002 significance compared to Post-treatment.

##### 3.2.3.4 Superoxide dismutase level

Results outlined in Figure 7d represent the depleted level of SOD in CRS rats compared to control (*p* < 0.001) rats. A minimal significant change was noted in animals that received only VCO compared to control (*p* = 0.02). An elevation was noted in VCO-treated Pre-VCO (*p* < 0.001), Co-VCO (*p* < 0.001), and Post-VCO (*p* < 0.001) groups compared to stress rats, signifying enhancement of SOD levels in hippocampus of the brain. Among the treatment groups, Pre-treatment exhibited (*p* < 0.001) significant elevation of SOD level compared to Co-treatment and Post-treatment, while Co-treatment exhibited *p* < 0.002 significance compared to Post-treatment.

##### 3.2.3.5 Catalase level

The histogram of CAT presented in Figure 7e outlines the detrimental effect of CRS on catalase level. Statistically, the CAT level was lowered in CRS rats compared to control rats (*p* < 0.001). A slightly significant change was noted in animals that received only VCO compared to control (*p* = 0.003). An upsurge was noted in the treated animals of Pre-VCO (*p* < 0.001), Co-VCO (*p* < 0.001), and Post-VCO (*p* < 0.001) groups compared to CRS rats, indicating that the CAT level in the hippocampus of the brain was amplified. Comparative analysis between the treatment groups showed that Pre-VCO demonstrated (*p* < 0.001) a significant elevation of CAT level compared to Co-treatment and Post-VCO, while Co-VCO demonstrated *p* = 0.003 significance compared to Post-treatment.

#### 3.2.4 Histopathology

##### 3.2.4.1 Histological changes in CA1, CA3, and the dentate gyrus region of the hippocampus

The Figure 8 represents the histopathological images of the CA1, CA3, and DG regions of the hippocampus stained with cresyl violet (Nissl staining). A clear distinction in neuronal cell morphology and organization was observed between CRS and the control group. The CRS group showed a higher number of darker cells (pyknotic neurons) in CA3 and DG regions of the hippocampus, accompanied by a scattered and distorted cellular arrangement, indicating neuronal damage. In contrast, the CA1 region of CRS rats showed no significant change in darker cells; however, the arrangement of neuronal cells was distorted when compared to both the control and VCO-only groups. Importantly, all three of the VCO-treated groups (Pre-VCO, Co-VCO, and Post-VCO) outperformed with notable improvement in CA3 and DG regions, with a reduced number of pyknotic cells and more organized cellular architecture compared to the CRS group.

**Figure 8:**
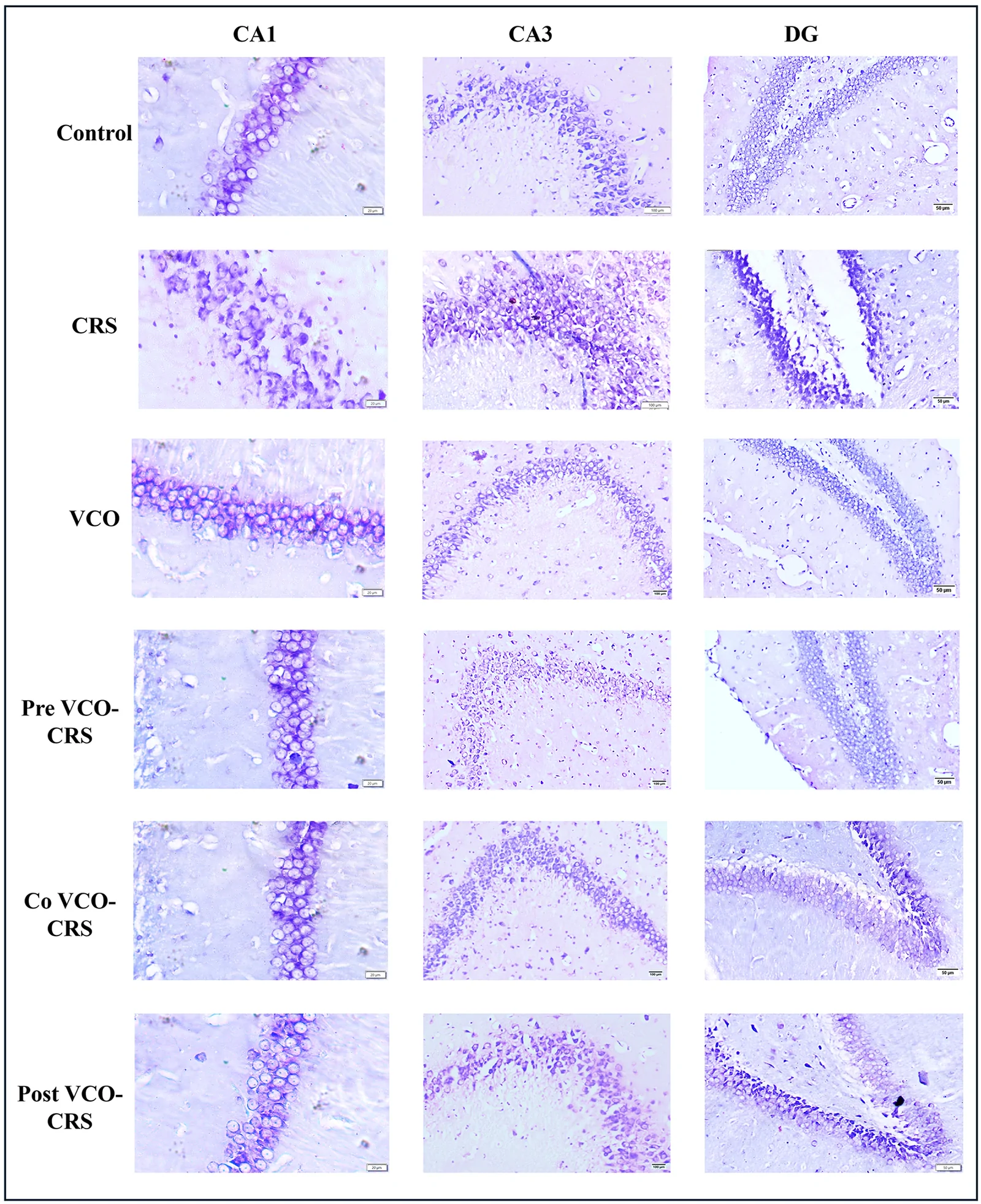
The effects of VCO on morphometric changes in the CA3 and Dentate gyrus (DG) region of the hippocampus. The CA1 images are captured at 40 × (20 μ) magnification, DG images are captured at 20 × (50 μ), and CA3 images are captured at 10 × (100 μ) magnification. We observed a distorted, scattered cell (CA1, CA3, & DG regions) and a visibly larger number of damaged cells (darker cells in CA3 and DG regions) in the CRS group. A close, dense, and even arrangement of neuronal cells was observed in the hippocampus (CA1, CA3, & DG) of VCO group rats.

##### 3.2.4.2 Histopathology of adrenal gland

Our current research depicts histopathological changes in the adrenal gland stained with H & E. The CRS rats exhibit stress-induced hypertrophy in the cortex region of the adrenal gland (Figure 9B). Hyperplasia (indicated with red arrows in Figure 9B) of cells of the adrenal cortex, along with the presence of lipoid deposits (indicated with blue arrows in Figure 9B), was also noted. Additionally, a separated, uneven (wavy) capsular layer (indicated with a green arrow) with the disturbed arrangement of zona glomerulosa cells and zona reticularis cells Figure 9B was observed. However, the VCO-treated groups Pre-VCO, Co-VCO, and Post-VCO shown in Figures 9D–F, respectively, demonstrated marked recovery, with improved structural features of the adrenal cortex compared to the CRS group. Furthermore, the suprarenal cortex in VCO only group (Figure 9C) appeared nearly normal and comparable to that of the control group (Figure 9A).

**Figure 9:**
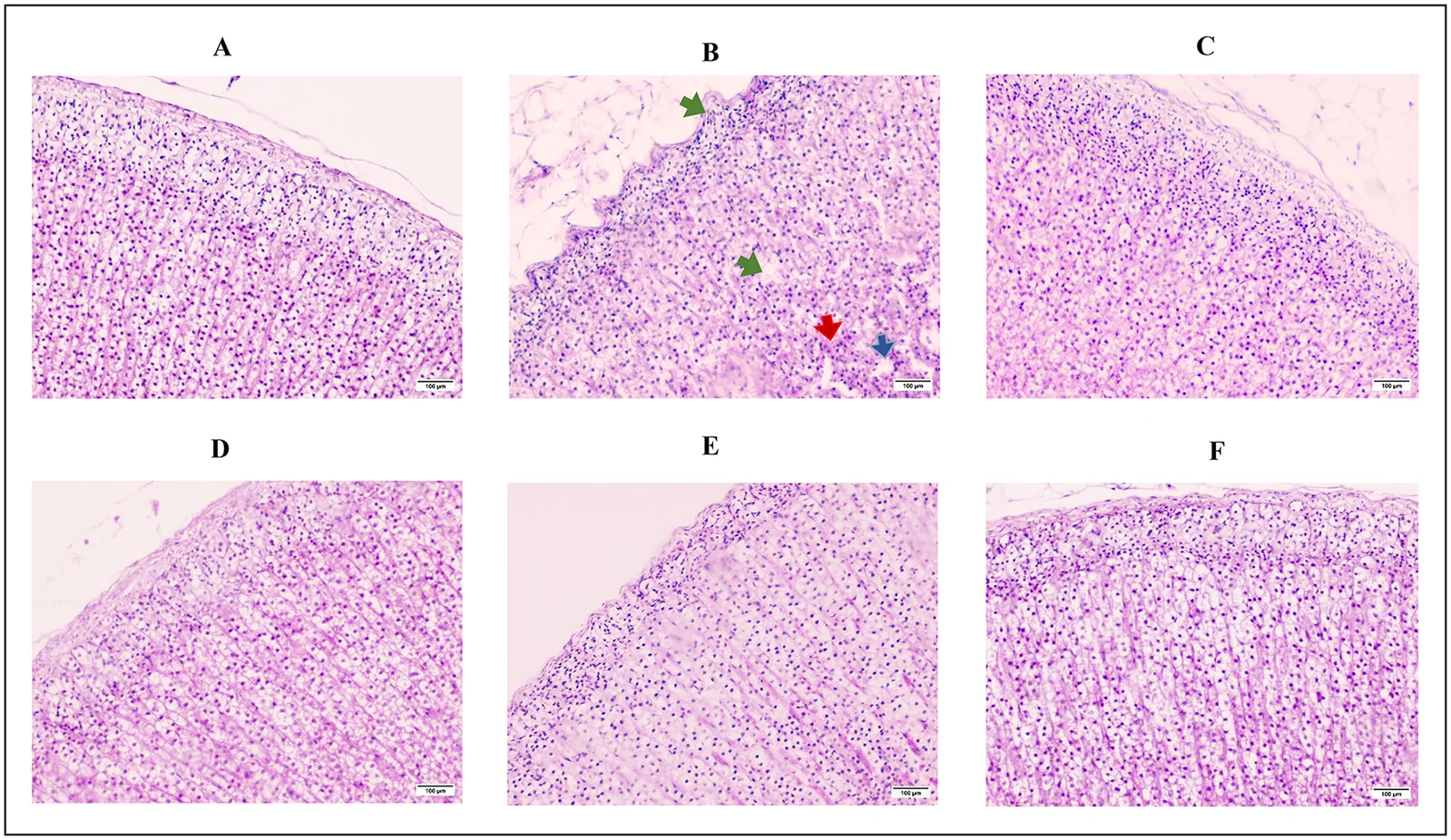
The effects of VCO on morphometric changes in the adrenal cortex of the rat induced by CRS, stained with hematoxylin and eosin. Image are represented as different group and denoted as Control **(A)**, CRS **(B)**, VCO only **(C)**, Pre VCO-CRS **(D)**, Co VCO-CRS **(E)**, Post VCO-CRS **(F)**. All the images are captured at 100 μ.

## 4 Discussion

### 4.1 GC-MS profiling of VCO

To investigate the core behind the pharmacological activity of VCO, we profiled the phytoconstituents in DVB and HLB columns of GC-MS. From all the metabolites traced in DVB and HLB, we found the highest peak of caprylic acid (Figure 3). On the contrary, many studies showed the highest peak of lauric acid in VCO composition (44). This may be due to differences in the coconut-growing region, as bioactive compounds vary in concentration by species, region, and plant part (45, 46). We interpret it as a region- and extraction technique-dependent variation rather than a deviation from VCO identity and quality (46, 47). Despite the intense crosstalk between bioactive peak intensities in GC-MS, we noticed an improvement in CRS rats. Undeniably, the presence of not only fatty acids like caprylic acid, myristic acid, caproic acid, and lauric acid, but also polyphenols and squalene, may have augmented the potency of VCO in the current study.

To date, numerous studies on the GC-MS analysis of VCO have primarily focused on MCFA and its function. In addition to previous reports (45), our GC-MS profiling revealed several unexplored metabolites of VCO (listed in Supplementary Tables 1, 2). Some phytocompounds appeared in smaller abundance; nonetheless, they may have a significant contribution to the neuroprotective activity of VCO. However, a detailed evaluation of region-specific changes in VCO composition is crucial to understanding the variation.

### 4.2 Enrichment analysis illustrates VCO's highest significance in the fatty acid metabolism pathway

To expand our mechanistic understanding of VCO activity, we performed an enrichment analysis from GC-MS data (Figure 2). It illustrated that VCO highest significance in the fatty acid biosynthesis pathway. Therefore, VCO serves as a rich source of MCFAs, which act as an alternative energy source in stressful conditions (23, 48–52). Our GC-MS data of VCO confirmed the dominance of caprylic acid, which rapidly converts into ketone bodies and supplies the highest amount of energy to the brain. And maybe assisting in regulating the cascade of reactions in the brain and reducing the workload of mitochondria in the energy-deficient CRS model (53–57).

Subsequently, VCO showed the highest impact on the Glycine serine threonine metabolism pathway and the second highest impact on the alanine aspartate and glutamate metabolism pathway (Figure 2). The Glycine serine threonine metabolism pathway has an interconnected role in brain neurotransmission, which is affected in CRS condition (8, 21, 51, 58). Another pathway participated in by VCO is the alanine-aspartate and glutamate metabolism pathway, which is central to amino acid turnover and energy metabolism and is tightly linked to glutamatergic neurotransmission (59). These findings may support our *in vivo* observations and may suggest that VCO may contribute to improving hippocampal function by normalizing glutamatergic metabolism. This interpretation is consistent with Edem et al. (22), who reported VCO-mediated modulation of hippocampal glutamatergic and GABAergic signaling. However, detailed molecular investigation is imperative to elucidate the exact pathway followed by VCO for attenuating CRS-induced hippocampal deformities.

### 4.3 *In vivo*- pharmacological assessment

CRS is one of the preclinical models utilized to replicate human psychological and emotional stress (5). In CRS, animals were deprived of freedom by immobilizing them in the same place for a long duration, mimicking the psychiatric condition in humans (6). Stress has a negative influence on the hippocampus and, consequently, affects the functioning of the body. The hippocampus plays a crucial role in the formation, consolidation, and retrieval of memory. Multimodal information from various brain regions converges in the hippocampus, primarily via the entorhinal cortex (EC).

### 4.4 VCO restores physiological alterations induced by CRS

Physiological alterations are the secondary changes that appear dominantly in the CRS condition and could be used as a sign to verify chronic stress (60). CRS activates the HPA axis, which augments the release of stress hormones (corticosterone and cortisol in rodents and humans, respectively) and signals an upsurge in lipolysis, glycogenolysis, and gluconeogenesis to compensate for the high energy demand, which impairs appetite. This leads to an augmentation in the catabolism of adipose tissue and muscle, ultimately contributing to a drop in the weight gain process (61). Similar results were evident in our study; animals exposed to CRS showed less weight gain. Analogous studies were reported on rats exposed to CRS, showing diminished animal weight gain (62, 63). However, a significant increase in weight gain was noticed during 21 days of VCO treatment administered in a time-staggered manner. This could be due to the modulatory action of VCO, which tightens the protein junctions of the gut barrier, improving integrity and retaining beneficial bacteria in the gut (64). This results in reduced systemic inflammation, thereby improving metabolic function and stabilizing the body weight gain process.

### 4.5 VCO restores adrenal gland weight and morphology

In CRS condition, the neuroendocrine system instigates the adrenal cortex to release high CORT, resulting in adrenal gland hypertrophy (17, 65). Our histological appearances are consistent with injury and confirm hypertrophy in CRS rats, with notable disruption of the adrenal cortex, including the capsule, zona glomerulosa, and zona reticulata. Chronic stress significantly elevates circulating CORT levels, which directly impacts adrenal gland morphology. Persistent activation of the HPA axis under CRS stimulates the adrenal gland to release excess corticosterone, resulting in adrenal hyperplasia. In our study, animals receiving VCO treatment showed histological appearances consistent with recovery, supported by a significant reduction in adrenal gland weight and serum CORT levels. Our results are consistent with previous studies demonstrating a strong correlation between elevated corticosterone levels and adrenal hypertrophy in chronic stress models (23). Collectively, these findings highlight the plausible role of VCO indirectly participating in CORT-assisted HPA axis regulation and may provide stress resilience.

### 4.6 VCO mitigate neurobehavioral impairment induced by CRS

The histological lesions often mirror the functionality of the hippocampus, and these changes can be most effectively evaluated through behavioral analysis. Basically, behavioral impairment is the foremost sign noticed in psychiatric patients. The individual with chronic stress responds minimally to surrounding activity, primarily due to the development of anhedonia (a diminished ability to experience pleasure), which is often attributed to anxiety disorders (66, 67). To address this behavioral sign in psychiatric patients in rodents, we performed SPT and EPM, respectively. SPT scored the degree of pleasure based on the consumption of sucrose, whereas EPM was employed for assessing the anxiety index. Our study indicates that CRS animals exhibited a decreased percentage of sucrose intake and an increased anxiety index. VCO treatment resulted in increased sucrose intake. A similar study by Wang et al. (68) on VCO reported similar expression of results, where VCO reduces anxiety symptoms (69, 70).

Damage to regions of the hippocampus triggered structural and functional destruction as well, apparently impairing learning, memory, and cognition in CRS-induced male rats (8). In our study, similar behavioral expressions were observed in a RAM, with CRS animals exhibiting forgetfulness during the task. The learning capability of CRS rats in the reward task was impaired, and they failed to recall the reward memory in the later period. On the contrary, VCO-treated rats recognized and memorized the clues kept on the baited arm and thereby showed significant improvement in learning ability. More specifically, VCO treatment has a more prominent effect on working memory than reference memory, though VCO attenuated the impaired working and reference memory of CRS rats. This indicates improved cognition, learning, and memory ability. Our study validates the previous reports and additionally documents the ameliorative effect of VCO in CRS-induced spatial learning and memory of the animal, which has been unraveled so far (71–73).

### 4.7 VCO remodulates the antioxidant status, dysregulated by CRS

The behavioral alterations are attributed to disrupting the cellular redox state, dendritic remodeling, inflammation, and immune response, and may lead to neuronal loss (74). In the current study, antioxidants and allied enzymes are significantly depleted in CRS rats compared to only VCO and Control group rats. These results are typically linked to the restraint stress-induced oxidative stress, probably due to the over-accumulation of ROS. Therefore, the primary target implemented to counter CRS is oxidative stress (75).

Previous studies exhibited that exogenous antioxidants have a remarkable enhancement in stress resilience and may restrict the progression of chronic stress-induced neurodegeneration (76, 77). In the current study, we treated CRS rats with naturally available antioxidant VCO (78). Treatment with VCO outperformed and showed improvement in levels of GSH, SOD, and CAT in the hippocampus of rats. More specifically, pretreatment with VCO showed an exceptional improvement over Co-VCO and Post-VCO treatment. As a result of the oxidation in neural tissue, MDA is produced in high concentration, which is considered another biomarker of CRS-induced oxidative stress. Likewise, in our study, the CRS group showed an amplified level of MDA. On the contrary, VCO administration reduced MDA concentration, which indicates a reduction in lipid peroxidation in neural tissue. Our study validates the previous research, indicating the potential of VCO highlighted by (72, 73, 79). This indicates the modulatory effect of VCO treatment, improving the balance of oxidants and the production of antioxidants and recuperating the oxidative stress damage.

### 4.8 VCO attenuates neuronal loss in the hippocampus induced by CRS

In preclinical research conducted by Aalling et al. (33) reported that CRS has a detrimental effect on the DG, predominantly and CA1 and CA3 of the hippocampus, causing dendritic shrinkage, synaptic plasticity with concomitant neuronal damage, which causes functional impairment with structural degradation. Likewise, in our present study, we observed several histological appearances consistent with injury in the hippocampi of CRS-induced rats. Neuronal cells in the DG, CA3, and CA1 regions stained deep blue or purple, with darker cells (damaged neurons) appearing irregular in shape, symbolizing pyknosis and cellular damage. Such damaged cells display ruptured membranes and disrupt cytoplasmic contents, allowing cresyl violet stain to diffuse throughout the cell. CRS arrests neural circuit processing and interrupts the functioning of the hippocampus. However, VCO-treated groups' appearances are consistent with recovery, exhibiting healthy neuronal morphology, which appeared purple-blue in color, showing distinct circular borders and a clearly visible nucleolus, suggesting intact rough endoplasmic reticulum and nuclear structures. This indicates that VCO supplementation facilitated neuronal restoration in the DG, CA3, and CA1 regions. Notably, all three treatment groups (Pre VCO-CRS, Co VCO-CRS, and Post VCO-CRS) offered equal amelioration in the CA1 region. However, Pre-treatment with VCO resulted in the most pronounced neuronal recovery in the DG and CA3 regions compared to Co-treatment and Post-treatment groups. This suggests that VCO may exert a neuroprotective effect by preconditioning neurons against the detrimental consequences of CRS, thereby mitigating hippocampal dysfunction and enhancing neuronal integrity. These findings also highlight the structural restoration and neuroprotective potential of VCO in the CA1, CA3, and DG subfields of the hippocampus.

In conclusion, VCO effectively attenuated the neurobiological consequences of CRS, supporting hippocampal structural and functional recovery, which may plausibly be mediated through the bioactivity of MCFAs, including caprylic acid, contributing to mitigation of oxidative stress and hippocampal neuronal dysfunction. Pre-treatment conferred the greatest neuroprotection, suggesting a prophylactic role whereby early administration may delay or prevent chronic stress-induced neurodegeneration. The observed improvements in Post-treatment group are consistent with, but not exclusively attributable to, a pharmacological effect of VCO, and may reflect endogenous post-stress recovery over the 21-day washout window. Integrating *in vivo* outcomes with GC-MS phytochemical profiling supports the positioning of VCO as a functional food nutraceutical with prophylactic and adjunctive neuroprotective efficacy, potentially attributable in part to its MCFA constituents, including caprylic acid. Further quantitative validation of VCO's fatty-acid composition using a validated method, alongside future clinical trials investigating VCO supplementation in stress-vulnerable populations, is warranted.

### 4.9 Limitation

This study is limited to the initial profiling of phytocompounds via untargeted GC-MS. Thus, a targeted GC-MS analysis would be a specific approach to enable precise quantification of individual phytochemicals. The *in-vivo* study warrants further consideration with larger pool of animals with a greater number of animals. Our design did not include a vehicle-only recovery arm (21 days CRS followed by 21 days vehicle without VCO), therefore we recommend a dedicated stress-then-vehicle recovery arm as a specific addition for future confirmatory work to identify the direct pharmacological effect of VCO in the Post-VCO group. Additionally, stereological quantification would strengthen these findings of hippocampal cells using Nissl staining would strengthen the study outcome. This will also, translation of this work into clinical trials should include physicochemical quality testing of VCO. Furthermore, molecular-level investigations are necessary to confirm the mechanisms underlying VCO's neuroprotective effects. To confirm the efficacy of nutraceuticals compared with traditional neuroprotective strategies, a clinical trial is warranted.

## Data Availability

The original contributions presented in the study are included in the article/Supplementary material, further inquiries can be directed to the corresponding author.
