# Supplementary material for: Timing-dependent neuroprotective effects of Virgin coconut oil against chronic restraint stress-induced neurobehavioral and hippocampal dysfunction: GC-MS profiling and in vivo evaluation
Source: Front Nutr. 2026 Oct 2;13:1936663. doi: 10.3389/fnut.2026.1936663 (PMC13672024; doi:10.3389/fnut.2026.1936663)
Supplement: Supplementary file 1 [file Supplementary_File_1.docx]

1. **GC-MS procedure**

**Procedure**

The Phytochemical profiling of VCO was carried out using Gas chromatography Mass spectroscopy (GC-MS) (GC/MS-QP2020 NX SHIMADZU, Shimadzu Corp., Tokyo, Japan) according to the previously published protocol of Poojary et.al. We performed thin-film solid-phase microextraction (TF-SPE) using two different disposable biodegradable paper-based double-coated patches: one with Divinylbenzene (DVB) sorbent and another with a hydrophilic-lipophilic balanced (HLB) sorbent. The extracted analyte was used for GC-MS analysis.

The procedure of GC-MS profiling is divided into 4 major steps. It begins with

**1.** **Patch preparation-** Here, we prepared 2 patches, Divinylbenzene (DVB) and HLB. The DVB patch was prepared according to Mandal *et.al* 2024. Briefly, a composite mixture of - a sorbent particle 1-5 µm (1 g DVB polymer) (Sigma-Aldrich), 4 mL polydimethylsiloxane (PDMS) (Sigma-Aldrich), and 0.06 mg multi-walled carbon nanotubes (Sigma-Aldrich) in 9 mL hexane was prepared. It creates a porous structure, allowing the adsorption of moderately polar metabolites of VCO(197).

For HLB patch preparation combination of Huang *et.al* 2023 and Grandy *et.al* 2018 procedure was followed. Briefly, the composite mixture of a HLB consists of a sorbent particle (DVB (lipophilic) + n-vinylpyrrolidone (hydrophilic) copolymer) 80%, PDMS 10 %, 0 multi-walled carbon nanotubes 5% in a solvent (methanol: acetone 1:1). The mixture was sonicated for 30-40 mins to form a uniform slurry. These materials are "balanced" between hydrophilic (water-attracting) and lipophilic (fat-attracting) properties, allowing the extraction of polar and non-polar molecules (198–200).

For both patches, the particle size of 1-5 µm was maintained in the mixture. To coat the particles, an eco-friendly, disposable and biodegradable thin-film of paper-based substrate (TF-PS) was used (a Copy Gold A4 sheet, GSM–75). The mixture was coated over a TF-PS using an automated applicator at a thickness of 70 µm on each side (to ensure the thickness, Elcometer 4340 was used). The TF-PS was dried in a controlled environment of nitrogen in a vacuum oven. The dried TF-PS sheet was further trimmed into small patches of measurement 4 cm x 1 cm, called as DVB patches. The prepared patches were stored in a sterile area away from contamination and moisture until next use.

**2.** **Microextraction of the analyte**- we performed solid phase microextraction using TF-PS. Prepared a novel thin film of paper, a double-coated DVB and HLB patch was used for microextraction. The patches, DVB and HLB were placed in a separate 20 ml glass vial containing 10 ml VCO while ensuring its completely immersed. The vial was sealed and placed into a shaking incubator (25 °C, 250 rpm) (REMI CIS-18 Plus). This allows the phytoconstituents of VCO to adsorb over the patch. After 60 minutes, the patch was removed carefully and placed in the desorption assembly.

**3.** **Desorption of analyte-** In this step, the adsorbed phytoconstituents of VCO were desorbed into the vehicle analyte. Here, acetonitrile (AN) was used as a vehicle analyte. The patches loaded with phytoconstituents of VCO (DVB and HLB) were dipped in a separate glass vial containing 1 ml of AN and allowed to vortex. After 60 minutes, the patches were removed from the analyte. Here, the desorption phase ends, leaving all the phytoconstituents in the analyte (AN). The obtained AN was ready for GC-MS analysis.

**4.** **GC-MS analysis of VCO**- Obtained acetonitrile and injected into GC-MS assembly. Concisely, the GC-MS (GC/MS-QP2020 NX SHIMADZU) consists of a column (SH-I-5Sil MS (30 m length, 0.25 mm internal diameter) flowing at a rate of 1.2ml/min and a carrier gas (helium) programmed at a specific condition (Initial 50°C (hold 2 min), ramp 6°C/min to 280°C (hold 1 min), ramp 15°C/min to 320°C (hold 2 min), ion source of 230 °C (EI) constant throughout the processing. All the samples were run in triplicate. This crucial step identifies the phytoconstituents present in VCO.

The metabolites of VCO passed through a column and entered in mass spectrometer, where the metabolites were fragmented by the ioniser and developed a chromatographic spectrum. The peak eluted at the relative retention time of the metabolite yields a mass spectrum, which facilitates the identification of phytoconstituents. The obtained metabolomic data were compared with the National Institute of Standards and Technology (NIST) library based on Component area, retention time, and mass spectral pattern for the profiling of phytoconstituents and cross-verified the composition with published GC-MS data of VCO (201,202). Further, the metabolomic data were used to explore the probable mechanistic pathway by performing enrichment analysis using MetaboAnalyst 6.0 software.

**Table 1. Details of phytocompounds identified in HLB**

| Sr. No. | Input name | Standardized name | Sub class | Component retention time | HMDB_ID | Component Area |
| --- | --- | --- | --- | --- | --- | --- |
| 1. | **Octanoic acid** | Caprylic acid | Saturated Fatty acids | 21.6171 | HMDB0000482 | 164663945.252 |
| 2. | **Dodecanoic acid** | Lauric acid | Saturated Fatty acids | 16.0481 | HMDB0000638 | 50031769.43 |
| 3. | **Acetamide** | Acetamide | Carboximidic acids | 6.44455 | HMDB0031645 | 27085929.110 |
| 4. | **delta-Dodecalactone** | Delta-dodecalactone | Fatty lactones | 14.844 | HMDB0037742 | 13037283.783 |
| 5. | **Decanoic acid** | Capric acid | Saturated Fatty acids | 12.9275 | HMDB0000511 | 12206204.057 |
| 6. | **Myristic acid** | Myristic acid | Saturated Fatty acids | 17.4051 | HMDB0000806 | 9933757.607 |
| 7. | **Glycerol tricaprylate** | Tricapyline | Saturated fatty acids | 23.02 | HMDB0011187 | 6676064.816 |
| 8. | **Octadecanoic acid** | Stearic acid | Saturated Fatty acids | 17.823 | HMDB0000827 | 6168061.075 |
| 9. | **Pentanoic acid** | Valeric acid | Saturated Fatty acids | 23.4112 | HMDB0000892 | 4427417.906 |
| 10. | **Thioglycolic acid** | Thioglycolic acid | Carboxylic acids | 6.47398 | HMDB0254464 | 2463077.825 |
| 11. | **n-Hexadecanoic acid** | Palmitic acid | Saturated Fatty acids | 16.5241 | HMDB0000220 | 2420016.720 |
| 12. | **Formic acid** | Formic acid | Carboxylic acids | 8.02058 | HMDB0000142 | 1980060.696 |
| 13. | **Hexanoic acid** | Caproic acid | Saturated Fatty acids | 23.1836 | HMDB0000535 | 1495716.256 |
| 14. | **Succinic acid** | Succinic acid | TCA acids | 14.6694 | HMDB0000254 | 1407495.062 |
| 15. | **Dodecanal** | Laurylaldehyde | Fatty aldehydes | 12.3232 | HMDB0033933 | 978234.468 |
| 16. | **Phthalic acid** | Phthalic acid | Hydroxybenzoic acids | 6.93928 | HMDB0002107 | 935362.275 |
| 17. | **Oxalic acid** | Oxalic acid | Dicarboxylic acids | 14.4196 | HMDB0002329 | 706094.562 |
| 18. | **d-Proline** | D-Proline | Amino acids | 21.5097 | HMDB0003411 | 375325.476 |
| 19. | **Supraene** | Squalene | C30 isoprenoids | 21.9931 | HMDB0000256 | 358516.815 |
| 20. | **2-Tridecanone** | 2-Tridecanone | Oxygenated hydrocarbons | 13.0503 | HMDB0034148 | 322162.236 |
| 21. | **2,5-Dihydroxybenzaldehyde** | Gentisate aldehyde | Hydroxybenzaldehydes | 9.1525 | HMDB0004062 | 321532.810 |
| 22. | **2,3-Butanediol** | 2,3-Butanediol | 1,2-diols | 6.58961 | HMDB0033007 | 299034.766 |
| 23. | **Pyridine** | Pyridine | Pyridine alkaloids | 6.4269 | HMDB0000926 | 276425.879 |
| 24. | **2-Butenoic acid** | Crotonic acid | Carboxylic acids | 11.0298 | HMDB0010720 | 229321.118 |

**Table 2. Details of phytocompounds identified in DVB.**

| Sr. No. | Input name | Standardized name | Sub class | Component Retention time | HMDB_ID | Component Area |
| --- | --- | --- | --- | --- | --- | --- |
| 1. | **Glycerol tricaprylate** | Tricapyline | Saturated fatty acids | 19.446 | HMDB0011187 | 436870309 |
| 2. | **Octanoic acid** | Caprylic acid | Saturated Fatty acid | 21.61118288 | HMDB0000482 | 136937347 |
| 3. | **Myristic acid** | Myristic acid | Saturated Fatty acid | 16.05997502 | HMDB0000806 | 37826291.03 |
| 4. | **l-Ascorbic acid** | Vitamin C | Gamma butyrolactones | 20.25710354 | HMDB0000044 | 33309952.92 |
| 5. | **delta-Dodecalactone** | Delta-dodecalactone | Fatty lactones | 14.84682507 | HMDB0037742 | 13407143.74 |
| 6. | **Dodecanoic acid** | Lauric acid | Saturated Fatty acid | 17.47244962 | HMDB0000638 | 12653814.72 |
| 7. | **1-Naphthoic acid** | 1-Naphthoic acid | Naphthalenes | 20.22927008 |  | 11558829.49 |
| 8. | **Octadecanoic acid** | Stearic acid | Saturated Fatty acid | 17.82359479 | HMDB0000827 | 10234406.03 |
| 9. | **n-Hexadecanoic acid** | Palmitic acid | Saturated Fatty acid | 16.52697898 | HMDB0000220 | 4789820.487 |
| 10. | **Decanoic acid** | Capric acid | Saturated Fatty acid | 12.11918689 | HMDB0000511 | 2359696.168 |
| 11. | **Cinnamaldehyde** | Cinnamaldehyde | Phenylpropanoids | 10.25401283 | HMDB0003441 | 1843933.307 |
| 12. | **4-Ethylbenzoic acid** | 4-Ethylbenzoic acid | Benzoic acids | 7.015157944 | HMDB0002097 | 1067315.783 |
| 13. | **Necrostatin-1** | - |  | 17.27275565 | HMDB0255500 | 1056512.225 |
| 14. | **Phthalic acid** | Phthalic acid | Hydroxybenzoic acids | 6.932108883 | HMDB0002107 | 524181.5469 |
| 15. | **Aspartic acid** | Aspartic acid | Amino acids | 8.815905532 | HMDB0000191 | 421243.3596 |
| 16. | **2-Octanone** | Octan-2-one | Oxygenated hydrocarbons | 9.212441653 | HMDB0031294 | 406924.9701 |
| 17. | **Tetradecane** | Tetradecane | Hydrocarbons | 21.06603903 | HMDB0059907 | 347362.7303 |
| 18. | **Orcinol** | Orcinol | Other phenols | 9.36621827 |  | 326671.6694 |
| 19. | **Formic acid** | Formic acid | Carboxylic acids | 8.987037328 | HMDB0000142 | 206797.7785 |
| 20. | **Glycine** | Glycine | Amino acids | 8.825611119 | HMDB0000123 | 194081.8389 |
| 21. | **Undecane** | Undecane | Hydrocarbons | 12.19122208 | HMDB0031445 | 193870.1228 |
| 22. | **6-Aminonicotinic acid** | Aminonicotinic acid | Nicotinic acid alkaloids | 9.270880114 | - | 180267.2805 |
| 23. | **2,4-Dihydroxybenzaldehyde** | 2,4-Dihydroxybenzaldehyde | Hydroxybenzaldehyde | 10.6959105 | HMDB0062484 | 171979.3073 |
| 24. | **2-butenoic acid** | Crotonic acid | Carboxylic acids | 13.66240287 | HMDB0010720 | 140255.8865 |
| 25. | **p-Toluic acid** | 4-Methylbenzoic acid | Hydroxybenzoic acids | 12.00502718 | HMDB0029635 | 124950.5348 |
| 26. | **2-Heptanone** | 2-Heptanone | Oxygenated hydrocarbons | 7.267343441 | HMDB0003671 | 116822.8907 |

*Red colour indicates common phytocompounds found in HLB and DVB.

**Fig.1.** Sucrose preference test model (created using BioRender)

**Fig.2.** Elevated Plus Maze

**Fig.3.** Radial arm maze
